# A non-invasive urinary diagnostic signature for diabetic kidney disease revealed by machine learning and single-cell analysis

**DOI:** 10.1371/journal.pone.0340096

**Published:** 2026-01-02

**Authors:** Yonggang Chen, Jintai Luo, Yingying Zheng, Xiaomei Jiang, Zixiang Yang, Xiaobing Liu

**Affiliations:** 1 Department of Urology, Loudi Central Hospital, Loudi, Hunan Province, China; 2 Department of Urology and Andrology, Minimally Invasive Surgery Center, Guangdong Provincial Key Laboratory of Urology, First Affiliated Hospital of Guangzhou Medical University, Guangzhou, Guangdong, China; 3 Loudi Hospital, First Affiliated Hospital of Guangzhou Medical University, Loudi, Hunan Province, China; 4 Fangchenggang Hospital of Traditional Chinese Medicine, Guangxi University of Chinese Medicine, Fangchenggang, Guangxi, China,; 5 Department of Cardiovascular Medicine, Loudi Central Hospital, Loudi, Hunan Province, China; Kwara State University, NIGERIA

## Abstract

**Background:**

Diabetic kidney disease (DKD) poses a significant health burden with inadequate diagnostic sensitivity. This study develops non-invasive biomarkers by integrating urinary and renal single-cell sequencing with machine learning.

**Methods:**

This study analyzed DKD single-cell and bulk transcriptomic data from public repositories. We established a computational pipeline to distinguish kidney-originating cells in urinary sediments, enabling the identification of injury-associated gene signatures. These signatures were refined using machine learning to develop a diagnostic model, which was validated in independent cohorts. The biomarkers were further verified in DKD renal tissues at single-cell resolution and across multiple nephropathies. Functional and spatial analyses confirmed biological relevance using transcriptomic and histological validation.

**Results:**

Single-cell analysis of 2,089 urine-derived cells identified eight renal cell types, including injured proximal tubule cells (Inj-PTC) showing upregulated injury markers (HAVCR1, VCAM1) and enriched apoptotic/TGF-β pathways. A machine learning-selected biomarker panel (PDK4, RHCG, FBP1) demonstrated strong diagnostic value (area under the curve, AUC > 0.9), with consistent downregulation across multiple chronic kidney diseases. PDK4 and FBP1 were specifically suppressed in DKD renal Inj-PTC (p < 0.05). Functional analyses revealed their involvement in glucose metabolic pathways, and their cell type-specific expression patterns were confirmed by transcriptomic and immunohistochemical data.

**Conclusions:**

This study identifies a three-gene biomarker panel (PDK4, RHCG, FBP1) as a promising non-invasive diagnostic tool for DKD. While demonstrating excellent diagnostic performance. It represents a tubular injury-associated gene signature that is detectable in urinary cells and shows strong association with DKD in transcriptomic datasets, presenting a promising candidate for a non-invasive diagnostic assay.

## Introduction

Diabetic kidney disease (DKD) is a prevalent and severe complication of diabetes [1,2], standing as a leading cause of end-stage renal disease worldwide and posing a substantial global health burden [3,4]. Current clinical diagnosis primarily relies on estimated glomerular filtration rate (eGFR) and proteinuria assessment, yet these conventional markers present significant limitations: eGFR alterations typically manifest only in advanced stages, while proteinuria demonstrates considerable variability and primarily reflects glomerular barrier dysfunction rather than specific tubular injury [5,6]. Although kidney biopsy remains the diagnostic gold standard, its invasive nature, associated complication risks, and high costs preclude routine clinical application [7].

The application of liquid biopsy in non-invasive disease diagnosis has gained significant prominence [8], with urinary biomarkers representing a particularly promising avenue due to their non-invasive accessibility and clinical utility [9,10]. While this field has yielded promising candidates like KIM-1, NGAL [11], and various transcriptomic markers [12,13], a fundamental challenge persists: the cellular heterogeneity of urine samples. The significant contamination by bladder and urethral epithelial cells often obscures the signal from kidney-derived cells, compromising the specificity and reliability of putative biomarkers [14].

We therefore hypothesized that integrating urinary single-cell transcriptomics with machine learning could overcome this heterogeneity issue and identify robust, renal cell-specific biomarkers for non-invasive DKD diagnosis. Single-cell RNA sequencing (scRNA-seq) technology is uniquely positioned to address this challenge, as it allows for the precise discrimination of distinct cell populations within complex samples like urine [14,15]. By applying scRNA-seq to urine sediments from DKD patients, we can directly characterize the transcriptomic profiles of renal cells, free from contaminating signals.

**Fig 1:**
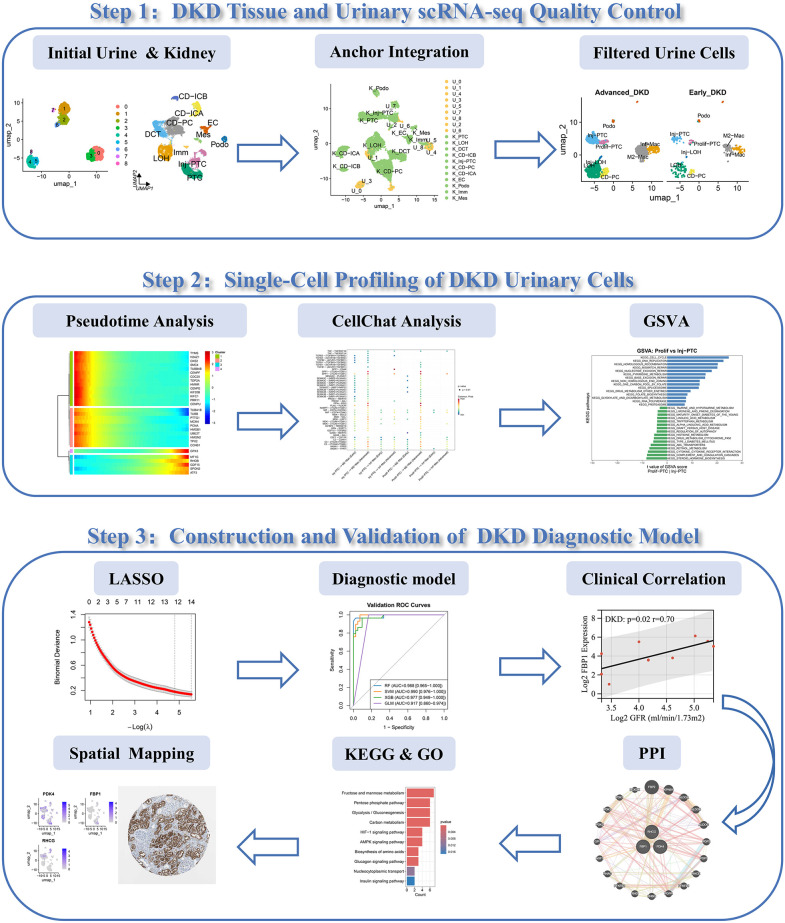
Single-Cell Profiling and Diagnostic Model Workflow for DKD. Abbreviations: scRNA-seq. Single-cell RNA sequencing; DKD, diabetic kidney disease; GSVA, Gene set variation analysis; LASSO, Least Absolute Shrinkage and Selection Operator; PPI, protein-protein interaction; KEGG, Kyoto Encyclopedia of Genes and Genomes; GO, Gene Ontology.

This study employed an integrated bioinformatics approach to systematically characterize molecular signatures in urinary single-cell sequencing data from DKD patients, with the primary aim of hypothesizing and preliminarily validating a novel non-invasive diagnostic signature and proximal tubule injury markers. As a hypothesis-generating investigation, our work sought to identify high-potential candidate biomarkers and lay a computational foundation for their future clinical translation. To this end, we constructed a detailed single-cell atlas of DKD urine sediments and renal tissues, implementing a computational pipeline to definitively distinguish kidney-originating cells from contaminants. We then characterized the functional states of proximal tubule cells and applied machine learning algorithms to develop and validate a combinatorial diagnostic model based on genes derived specifically from renal cells. The biological relevance of the identified biomarkers was further elucidated through protein-protein interaction network analysis and functional enrichment, with their spatial localization confirmed using external datasets. These findings provide novel insights for the future development of non-invasive diagnostic tools and targeted therapeutic strategies. The research flowchart is shown in **Fig 1**.

## Materials and methods

### Data access and pre-processing

Transcriptomic datasets were acquired from the Gene Expression Omnibus (GEO) database. The analysis included bulk RNA-seq datasets from GSE96804 (40 DKD patients and 21 healthy donors), GSE104948 and GSE104954 (collectively 30 DKD patients and 42 controls), and GSE142025 (27 DKD patients and 9 controls). For single-cell analysis, we utilized three datasets: GSE131882 provided kidney tissue profiles from 3 DKD patients and 3 controls; GSE266146 contained urine sediment data from 4 early-stage (eGFR ≥ 30 mL/min/1.73 m²) and 4 late-stage (eGFR < 30 mL/min/1.73 m²) DKD patients; GSE157640 contributed urinary single-cell data from 10 healthy individuals. All data analyzed were publicly available and de-identified, and the authors had no access to information that could identify individual participants. The original studies were conducted under ethical standards and obtained appropriate participant consent; no additional ethics approval or consent was required. Genes matching multiple probes were processed by averaging their expression values, with all dataset characteristics verified against original publications through the GEO portal (https://www.ncbi.nlm.nih.gov/geo/, accessed January 8, 2025). The integrated study design illustrating dataset utilization across different analytical phases is schematically summarized in **S1 Fig**.

### DKD tissue and urinary scRNA-seq quality control

Single-cell RNA sequencing data from DKD renal tissues and urinary sediments underwent comprehensive quality control using Seurat (v5.3.0). Cells were filtered based on established thresholds for gene counts and mitochondrial content to remove low-quality cells and doublets. Batch effects were corrected using Harmony (v1.2.3), with expression data normalized and scaled following standard single-cell processing workflows. Dimensionality reduction was performed using principal component analysis followed by UMAP visualization. Cell types were annotated based on canonical markers from established renal single-cell references [14,16–19].

An anchor-based integration method was used to align urinary and renal datasets, enabling identification and removal of bladder/urethral epithelial contaminants [14,20,21]. The integrity of renal cell populations was maintained throughout this process, ensuring reliable downstream analysis. Complete methodological details including quality control thresholds, integration parameters, and contaminant identification criteria are provided in the Supplementary Methods.

### Single-cell analysis of DKD urinary subpopulations

After excluding cells contaminated from the bladder/urethra, we re-performed quality control and dimensionality reduction clustering on the remaining cells according to the standardized workflow described above. We annotated the cell types of the DKD urine sediment single-cell subpopulations using recognized marker genes [14,22] and the CellMarker [23] website. Based on the annotated cell subpopulations, we employed the Monocle software package (v2.36.0) to construct pseudo-temporal trajectories [24–26]. This process involved dimensionality reduction followed by computational ordering of cell states along inferred trajectories.

To analyze intercellular communication networks between different cell subpopulations, we applied CellChat (v2.2.0) to systematically assess ligand-receptor pair expression [27]. For functional characterization, we performed Gene Set Variation Analysis (GSVA) using the GSVA package (v2.2.0) to quantify pathway enrichment differences between specific cell subpopulations. The analysis utilized Hallmark and Kyoto Encyclopedia of Genes and Genomes (KEGG) pathways retrieved from the Molecular Signatures Database [28]. While healthy control urine sediments were initially processed, these samples exhibited predominant bladder/urethral cell contamination and were consequently excluded from downstream analyses Finally, we utilized the FindMarkers function to compare transcriptomic profiles between advanced and early-stage DKD cells, with differentially expressed genes (DEGs) selected under the criteria of adjusted P < 0.05 and |logFC| > 1.

### DKD diagnostic signature development and validation

We implemented a multi-stage analytical framework to establish a robust diagnostic signature for DKD while controlling for overfitting. Differential gene expression analysis from urinary single-cell data informed our initial feature selection. We applied LASSO regression using the glmnet package (v4.1.9) exclusively on the training cohort GSE96804 (40 DKD vs 21 controls), identifying 14 candidate genes through 10-fold cross-validation. To enhance generalizability, we evaluated these candidate genes in an independent validation cohort (combined GSE104948/GSE104954; 30 DKD vs 42 controls) using ROC analysis (pROC v1.18.5). Only genes achieving an area under the curve (AUC) > 0.8 were retained, yielding a refined three-gene panel (PDK4, RHCG, and FBP1).

Using this finalized gene set, we constructed multivariate diagnostic models through four machine learning algorithms in caret (v 7.0.1): random forest (RF; randomForest v4.7.1.2), support vector machine (SVM; kernlab v0.9.33), extreme gradient boosting (XGBoost; xgboost v1.7.11.1), and logistic regression (LR). Model training employed stratified repeated 5-fold cross-validation (3 repeats) with specific tuning strategies: automatic mtry optimization for RF, sigma and C grid search for SVM, and fixed parameters (max_depth = 3, eta = 0.1) for XGBoost. The three-gene panel was fixed prior to model building and not re-selected during cross-validation, ensuring validation of a stable biomarker signature.

The locked models were applied to a completely held-out validation cohort B (GSE142025; 27 DKD vs 9 controls) for unbiased performance assessment. Area under the curve (AUC) with 95% confidence intervals served as the primary metric. For clinical translation, we developed a nomogram using the rms package (v8.0.0) and performed decision curve analysis (rmda v1.6) to evaluate clinical utility across probability thresholds [29]. This comprehensive approach balanced methodological rigor with practical biomarker development requirements.

### Clinical correlation analysis

To evaluate the clinical relevance of the identified biomarkers, their expression levels were analyzed using the Nephroseq v5 database (http://v5.nephroseq.org). Pearson correlation analysis was performed to assess the relationship between biomarker expression and estimated glomerular filtration rate (eGFR). The eGFR values were calculated using the Modification of Diet in Renal Disease (MDRD) equation. Additionally, we compared biomarker expression levels across multiple patient cohorts and healthy controls using t-tests. The cohorts included patients with minimal change disease (MCD), lupus nephritis (LN), focal segmental glomerulosclerosis (FSGS), IgA nephropathy (IgAN), and membranous glomerulonephritis (MGN).

### Molecular network construction and functional enrichment analysis

To investigate the protein interaction network of DKD-related biomarkers, we utilized GeneMANIA (http://genemania.org). This platform integrates multi-omics data—including physical interactions, co-expression, genetic interactions, and pathway associations—to predict functional linkages. By applying machine learning algorithms, GeneMANIA optimizes data weights to construct PPI networks and predict functionally linked genes. To further characterize the biological roles of these biomarkers and their associated genes, we performed KEGG pathway and Gene Ontology (GO) enrichment analyses. A false discovery rate (FDR)-corrected p-value < 0.05 was set as the threshold for statistical significance. The KEGG analysis identified key metabolic and signaling pathways, while the GO analysis systematically annotated gene functions across three categories: biological processes, molecular functions, and cellular components.

### Spatial biomarker mapping via scRNA-seq and IHC

To characterize the spatial expression profiles of diagnostic biomarkers, an integrated approach combining scRNA-seq data with immunohistochemical (IHC) validation was implemented. The single-cell transcriptomic dataset GSE131882 derived from DKD kidney tissues was analyzed using Seurat software to delineate biomarker expression patterns across renal cell populations. In parallel, immunohistochemical data corresponding to these biomarkers were retrieved from the HPA database (https://www.proteinatlas.org/) to corroborate their protein-level localization within kidney tissue architecture.

### Statistical analyses

All statistical analyses were conducted in R (v 4.5.1). A two-sided P-value < 0.05 was considered significant. Data distribution and variance homogeneity were assessed (Shapiro-Wilk/Levene’s tests) to guide test selection: continuous variables used t-test (two groups) or ANOVA (multi-group), or their non-parametric equivalents (Wilcoxon rank-sum test, Kruskal-Wallis); categorical variables used Chi-square/Fisher’s exact tests. Post-hoc tests followed significant multi-group findings. Correlations with eGFR used Pearson’s method; diagnostic model performance is reported as AUC (95% CI).

Multiple testing corrections were applied as appropriate. An FDR-adjusted p-value < 0.05 was used for differential gene expression analysis and all functional enrichment analyses (GSVA, KEGG, GO). Within the Nephroseq cohort, pre-planned pairwise comparisons between DKD and other CKD types employed Bonferroni correction. In contrast, p-values from other analyses are reported as nominal p-values. This applies to the cell proportion comparisons and to the series of independent hypothesis tests, each designed to evaluate a single disease against controls.

## Results

### Single-cell profiling of kidney cells in DKD

**Fig 2:**
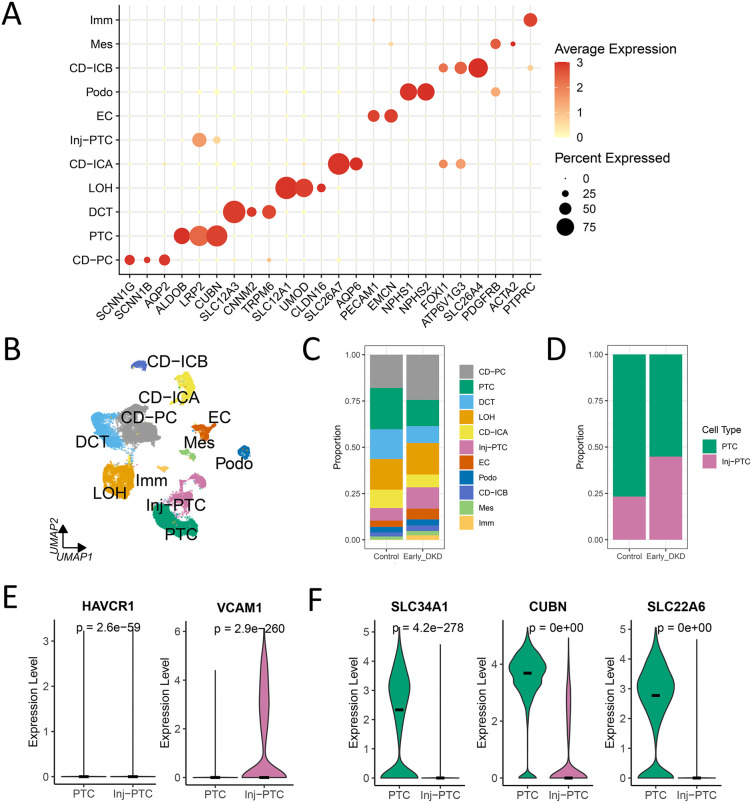
Single-cell profiling of the human kidney in DKD. **(A)** Dot plot displaying the expression of canonical marker genes used to identify the 11 major renal cell types. **(B)** UMAP projection of 20,220 high-quality cells from the integrated dataset (GSE131882; n = 3 controls, n = 3 DKD patients), color-coded by annotated cell type. Unsupervised clustering identified 11 distinct renal cell subpopulations, with key types abbreviated as follows: CD-PC (collecting duct principal cell); PTC (proximal tubule cell); Inj-PTC (injured PTC); DCT (distal convoluted tubule cell); LOH (loop of Henle); CD-ICA/CD-ICB (collecting duct intercalated cells); EC (endothelial cell); Podo (podocyte); Mes (mesenchymal cell); Imm (immune cell). **(C)** Stacked bar plot showing the proportional abundance of each cell type in control and DKD groups. **(D)** Stacked bar plot comparing the proportions of injured proximal tubule cells (Inj-PTC) and normal PTCs between control and DKD groups. The difference in proportion was assessed using a Chi-square test for categorical variables, and the p-value is unadjusted. **(E-F)** Violin plots showing the expression levels of injury markers **(E)** HAVCR1 and VCAM1, and functional genes **(F)** SLC34A1, CUBN, and SLC22A6 in Inj-PTC (n = 1794) versus normal PTC (n = 3794) clusters. Differential expression analysis for each gene was performed using the Wilcoxon rank-sum test; p-values are unadjusted. The black horizontal bar within each violin represents the median expression value.

We performed an integrated analysis of renal single-cell transcriptome data (GSE131882) from 3 DKD patients and 3 healthy controls, yielding 20,220 high-quality cells. Unsupervised clustering and marker-based annotation identified 11 distinct renal cell subpopulations, including collecting duct principal cells (CD-PC), proximal tubule cells (PTC), injured proximal tubule cells (Inj-PTC), distal convoluted tubule cells (DCT), loop of Henle (LOH), collecting ductal interstitial A/B-type cells (CD-ICA/CD-ICB), endothelial cells (EC), podocytes (Podo), mesenchymal cells (Mes), and immune cells (Imm) (**Fig 2A and 2B**). Notably, proximal tubular cells in DKD exhibited pronounced heterogeneity, with stacked histograms revealing a significant expansion of Inj-PTC proportions compared to controls (p < 0.05) (**Fig 2C and 2D**). Consistent with this, Inj-PTC demonstrated upregulated expression of injury markers (HAVCR1, VCAM1; p < 0.05) alongside downregulation of tubular functional genes (SLC34A1, CUBN, SLC22A6; p < 0.05) (**Fig 2E and 2F**).

### Single-cell mapping of renal-origin urinary cells in DKD

**Fig 3:**
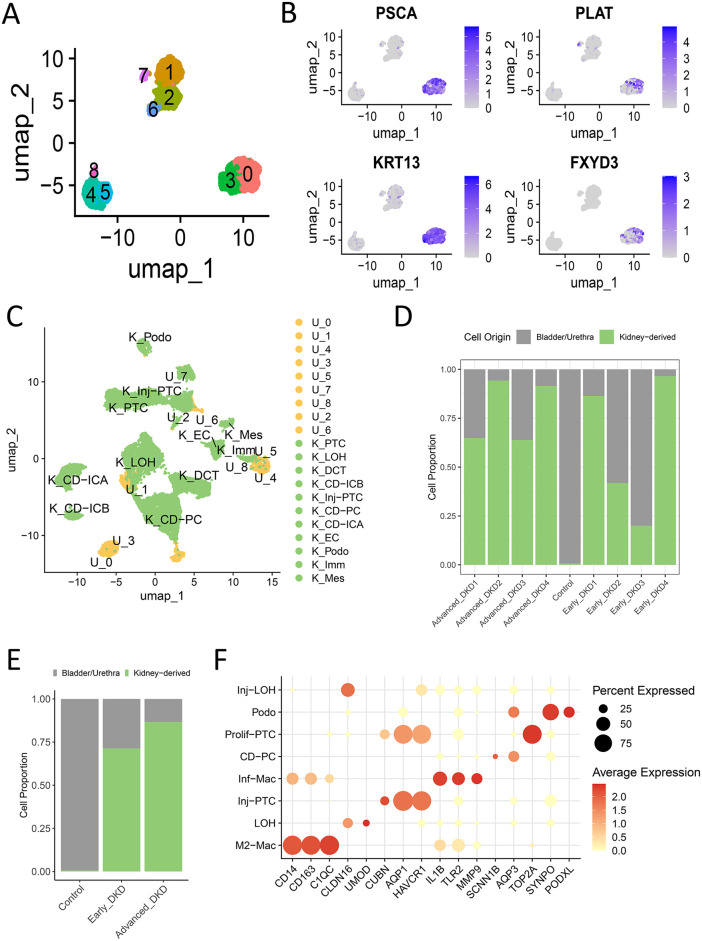
Identification and exclusion of non-renal cells in DKD urinary sediment single-cell data. **(A)** UMAP projection of 3,421 cells from DKD urinary sediments (GSE266146) and healthy controls (GSE157640), colored by initial unsupervised clustering. The 9 clusters (labeled 0-8) represent groups of cells with similar gene expression profiles, identified computationally prior to biological annotation. **(B)** Feature plots demonstrating the expression of bladder/urethral epithelial markers (PSCA, KRT13, FXYD3, PLAT) in specific clusters. **(C)** Integrated UMAP projection combining cells from kidney tissue (green) and urinary sediment (yellow). Kidney tissue cells (green, from GSE131882) are pre-annotated with “K_” prefixes (e.g., K_Podo for podocytes). Urinary sediment cells (yellow) are labeled with “U_” prefixes and cluster numbers, showing their spatial relationship to known kidney cell types. **(D-E)** Stacked bar plots illustrating the proportion of contaminating (bladder/urethral) cells versus renal-origin cells in the urinary sediments of control, early-stage DKD, and late-stage DKD groups. **(F)** Dot plot of canonical marker genes used to annotate the renal cell types within the urinary sediment data after quality control and the exclusion of non-renal clusters.

We integrated single-cell transcriptomes from urine sediments of 4 early-stage DKD, 4 late-stage DKD patients (GSE266146), and 10 healthy controls (GSE157640). The 3,421 urinary cells were classified into nine distinct subpopulations. Clusters 0 and 3 exhibited high expression of bladder/urethral epithelial markers (KRT13, PLAT, FXYD3, PSCA; **Figs 3A and 3B**). This was confirmed through anchor-based integration with renal tissue data (GSE131882), where these clusters (yellow) showed clear spatial separation from renal-derived cells (green) in UMAP projections (**Fig 3C**). In contrast, other urinary subpopulations were spatially closely associated with renal cell clusters. Crucially, subsequent analysis revealed that the urine sediments from healthy controls were predominantly composed of these bladder/urethral epithelial cells (clusters 0 and 3), with a negligible proportion of renal-origin cells (**Figs 3D and 3E**). Therefore, the non-renal clusters (0 and 3, totaling 1,332 cells) were excluded from all downstream analyses.

**Fig 4:**
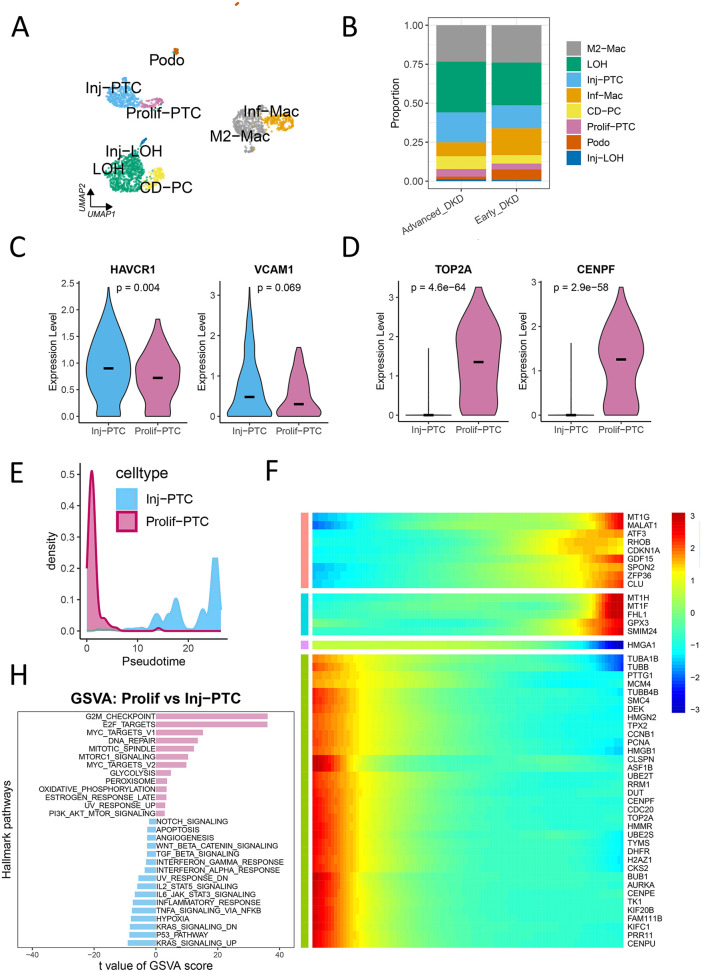
Characterization of renal cell subtypes in DKD urinary sediments. **(A)** UMAP projection of 2,089 high-quality, kidney-derived cells from urinary sediments of DKD patients (n = 4 early-stage, n = 4 late-stage). Cells are color-coded by annotated type. Key populations include injured and proliferative proximal tubule cells (Inj-PTC, prolif-PTC), podocytes (PODO), loop of Henle cells (LOH, Inj-LOH), collecting duct principal cells (CD-PC), and macrophage subtypes (M2-Mac, Inf-Mac). **(B)** Stacked bar plot depicting the relative abundance of each annotated renal cell type in early and late stages of DKD. **(C-D)** Violin plots comparing the expression of injury markers **(C)** HAVCR1 and VCAM1, and proliferation markers **(D)** TOP2A and CENPF between Inj-PTC (n = 385) and prolif-PTC (n = 98) clusters. The black horizontal bar represents the median expression value. Differential expression for each gene was performed using the Wilcoxon rank-sum test; p-values are unadjusted. **(E-F)** Transcriptional dynamics during proximal tubule injury. **(E)** Pseudotime ordering of cells from a proliferative (Prolif-PTC, purple) to an injured state (Inj-PTC, blue). **(F)** Corresponding heatmap shows gene expression changes (blue: low; red: high) along this trajectory, revealing genes activated or suppressed during injury. **(G)** Gene Set Variation Analysis (GSVA) displaying pathways enriched in prolif-PTC (top, purple) and Inj-PTC (bottom, blue) clusters.

Re-analysis of the remaining 2,089 high-quality, renal-origin cells (all derived from the 8 DKD patients) identified eight renal cell types: collecting duct principal cells (CD-PC), proliferative proximal tubule cells (prolif-PTC), injured proximal tubule cells (Inj-PTC), normal and injured loop of Henle (LOH, Inj-LOH), podocytes (PODO), M2-like macrophages (M2-Mac), and inflammatory macrophages (Inf-Mac), as visualized in **Figs 3F and 4A**. Subsequent analyses were conducted exclusively on these renal-origin cells from DKD patients.

### Urinary single-cell atlas of proximal tubule injury in DKD

Stacked histogram analysis displayed the proportional distribution of renal cell types, including Inj-PTC, in urinary sediments from control, early-stage, and advanced DKD patients **(Fig 4B)**. Quantitative analysis demonstrated marked upregulation of the tubular injury marker HAVCR1 (p = 0.004) and a non-significant trend for VCAM1 increase (p = 0.069) in Inj-PTC relative to prolif-PTC (**Fig 4C**). Conversely, proliferation markers (TOP2A, CENPF) showed significant downregulation in Inj-PTC (p < 0.05, **Fig 4D**). Pseudotime trajectory analysis delineated a transcriptional transition from prolif-PTC to Inj-PTC. Along this trajectory, gene expression shifted from proliferation-related markers (TOP2A, CENPF, TPX2) in early-stage cells to injury-associated markers (MT1G, GDF15, CDKN1) (**Figs 4E and 4F**). GSVA pathway analysis confirmed distinct molecular profiles: Inj-PTC exhibited activation of apoptosis, TGF-β signaling, hypoxia response, and inflammation, while prolif-PTC maintained DNA repair and energy metabolism pathways (**Fig 4G**). In macrophage subpopulations, pseudotime analysis revealed progressive upregulation of inflammatory markers (MMP7, MMP9, IL1B, S100A8) during M2-mac to Inf-mac transition (**S2****A and** **S2B Figs**). CellChat analysis identified SPP1-(ITGA4 + ITGB1)-mediated tubule-macrophage crosstalk exclusively in late-stage DKD (**S2C Fig**).

### Machine learning discovers diagnostic biomarkers for DKD

**Fig 5:**
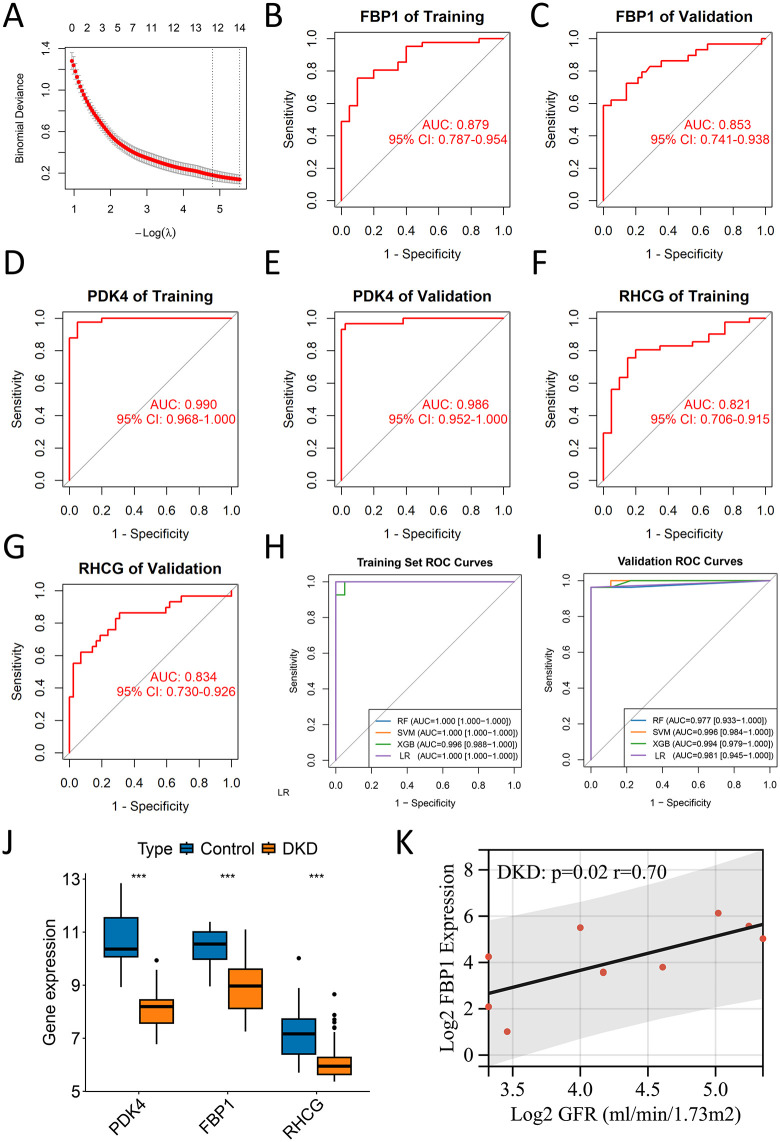
Machine learning-based discovery and validation of diagnostic biomarkers for DKD. **(A)** LASSO regression coefficient profile of genes differentially expressed in early vs. advanced DKD urinary cells. **(B-G)** Receiver operating characteristic (ROC) curves evaluating the diagnostic performance of PDK4, RHCG, and FBP1 in distinguishing DKD from controls in the training set (n = 40 DKD, 21 controls) and independent validation sets (n = 30 DKD, 42 controls). (H-I) Performance evaluation of multi-gene diagnostic models built using four machine learning algorithms in the training set (H) and independent validation set (n = 27 DKD, 9 controls) **(I)**. **(J)** Box plots showing the expression levels of PDK4, RHCG, and FBP1 in control versus DKD groups (n = 21 controls, 40 DKD). Statistical significance was determined using the Wilcoxon rank-sum test; p-values are unadjusted. (*P < 0.05, **P < 0.01, ***P < 0.001). **(K)** Scatter plot with regression line showing the correlation between FBP1 expression and estimated glomerular filtration rate (eGFR) in DKD patients from the Nephroseq database.

A machine learning workflow identified 14 candidate genes from urinary sediment single-cell data of DKD patients. Subsequent validation in an independent cohort (GSE104948/GSE104954) refined this to a core three-gene signature (PDK4, RHCG, and FBP1), which demonstrated high diagnostic accuracy (AUCs > 0.8) in distinguishing DKD from controls (**Fig 5A-5G**, **S1 Table**). Expression of these three genes was significantly lower in DKD compared to controls in the validation set (**Fig 5J**). However, the remaining 11 genes showed lower diagnostic efficacy (**S3****A and** **S3B Figs**, **S1 Table**).

On the completely held-out validation cohort (GSE142025), the locked three-gene models achieved AUCs of 0.977–0.996 (**Fig 5H and 5I**, **S2 Table**). The clinical utility of the signature was further supported by a nomogram and decision curve analysis (**S3****C and** **S3D Figs**). When comparing Inj-PTC to prolif-PTC in DKD patient urine, expression levels of FBP1 and PDK4 were significantly elevated (p < 0.05), whereas RHCG expression showed no significant difference (**S3E Fig**).

### PDK4/FBP1 downregulation mirrors PTC injury in DKD tissues

**Fig 6:**
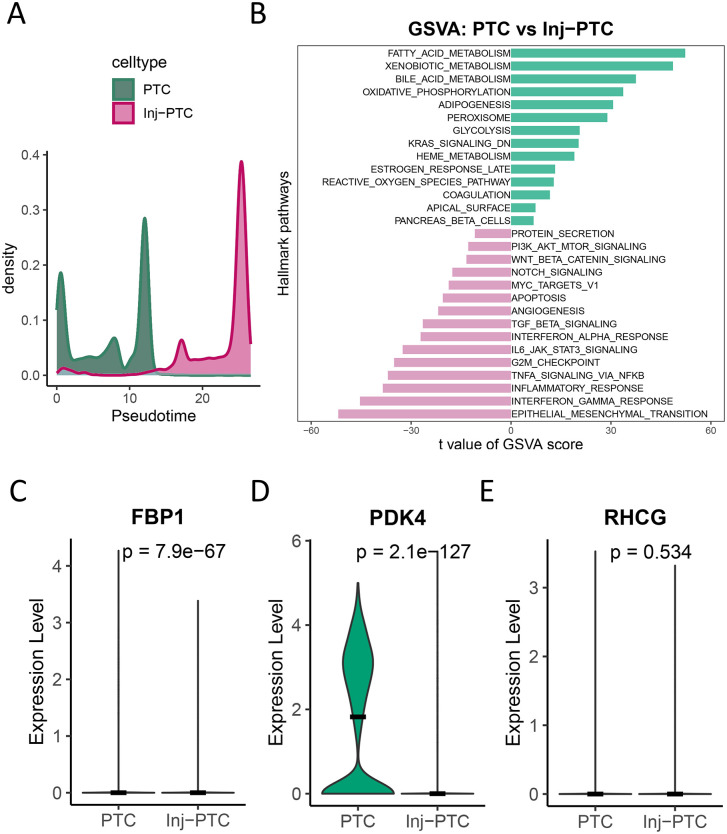
Expression patterns of diagnostic biomarkers in DKD kidney tissues. **(A-B)** Pseudotime and pathway analysis of kidney PTCs, GSVA enrichment scores showing pathways upregulated in normal PTC (top, blue) and Inj-PTC (bottom, blue).

Building upon urinary sediment findings, we characterized diagnostic marker expression in DKD kidney tissues. GSVA revealed significant enrichment of apoptosis, TGF-β signaling, inflammatory response, and epithelial-mesenchymal transition pathways in the Inj-PTC of DKD kidneys (**Fig 6A and 6B**). This transcriptional pattern mirrored that observed in Inj-PTC from urinary sediments.. Notably, PDK4 and FBP1 exhibited marked downregulation in kidney Inj-PTC versus normal PTCs (p < 0.05, **Figs 6C and 6D**); however, RHCG expression showed no significant difference (p = 0.534, **Fig 6E**). Bulk transcriptome data from the Nephroseq database demonstrated downregulation of these genes not only in DKD but also in FSG, IgAN, LN, MCD, and MGN (p < 0.05; **S4 Fig**). Further analysis revealed that while RHCG exhibited distinct expression patterns in DKD compared to other nephropathies, FBP1 and PDK4 showed comparable expression levels across disease types **(****S5 Fig****)**. Clinical correlation analysis highlighted a strong positive association between FBP1 expression and eGFR (r = 0.70, p = 0.02).

(C-E) Violin plots showing the expression of candidate diagnostic genes (C) FBP1, (D) PDK4, and (E) RHCG in Inj-PTC (n = 1794) versus normal PTC (n = 3794) clusters from kidney tissue data. Differential expression for each gene was performed using the Wilcoxon rank-sum test; p-values are unadjusted. The black horizontal bar represents the median expression value.

### Spatial expression and functional networks of DKD diagnostic markers

**Fig 7:**
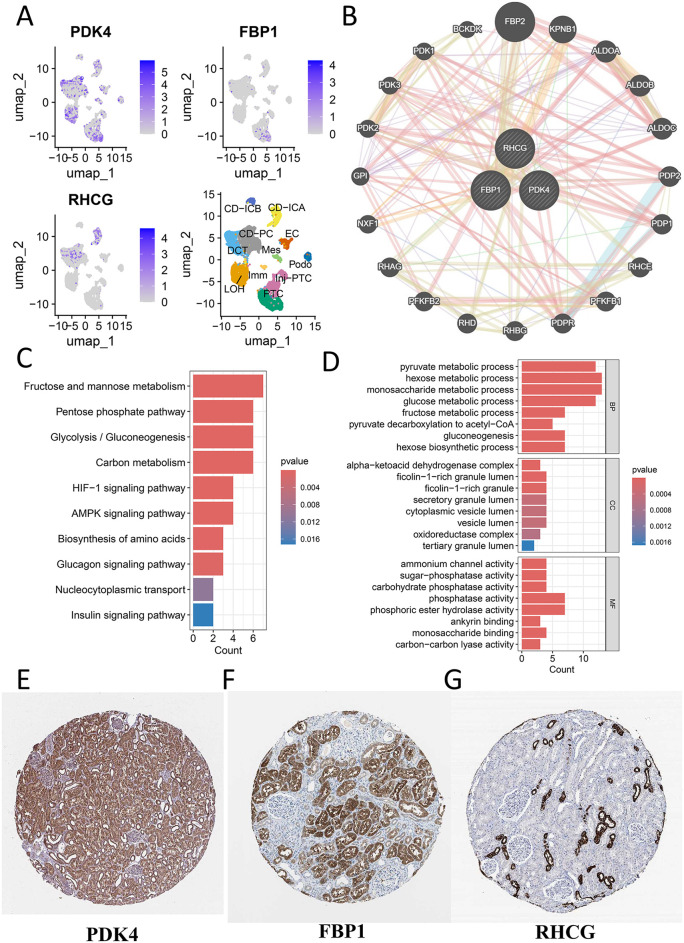
Spatial localization and functional enrichment of DKD diagnostic markers. **(A)** UMAP projection of DKD kidney single cells (GSE131882), with feature plots showing the expression patterns of PDK4, RHCG, and FBP1. **(B)** Protein-protein interaction (PPI) network of the three hub genes and their co-expressed genes. **(C)** Bar plot of the top enriched KEGG pathways for the gene network. **(D)** Bar plot of the top enriched Gene Ontology (GO) terms, categorized into Biological Process (BP), Cellular Component (CC), and Molecular Function (MF). **(E-G)** Representative immunohistochemistry images from the Human Protein Atlas (HPA) database showing the protein expression of **(E)** PDK4, **(F)** FBP1, and **(G)** RHCG in normal human kidney tissue.

Single-cell analysis revealed distinct spatial expression patterns of the three biomarkers: PDK4 was broadly distributed across renal tubular epithelia, whereas FBP1 exhibited specific expression in PTC but not in Inj-PTCs, and RHCG localized to distal tubules and collecting ducts (**Fig 7A**). These findings were further validated by immunohistochemistry from the HPA database, showing strong concordance with transcriptomic data (**Fig 7E-7G**). Gene interaction network analysis identified 20 co-expressed genes associated with PDK4, FBP1, and RHCG (**Fig 7B**). Functional enrichment demonstrated their involvement in key metabolic pathways, including glycolysis/gluconeogenesis, HIF-1 signaling, and insulin/glucagon regulation (KEGG; **Fig 7C**). At the molecular level, GO analysis highlighted their roles in gluconeogenesis, pyruvate metabolism, and energy conversion, with their products localized to secretory granules and oxidoreductase complexes (**Fig 7D**).

## Discussion

DKD remains a major clinical challenge due to the limited sensitivity of conventional biomarkers for detecting early tubular injury. Our integrated analysis enabled the discovery of three candidate diagnostic markers for DKD, including PDK4, RHCG, and FBP1. The validation cohort showed strong diagnostic performance, with individual markers achieving AUCs of 0.834–0.986 and the combined model reaching 0.977–0.996. At the cellular level, we observed significant downregulation of PDK4 and FBP1 in Inj-PTC. Importantly, this downregulation pattern was not unique to DKD but was consistently observed across multiple nephropathies. These damaged cells displayed characteristic activation of multiple pathological pathways, including TGF-β signaling, apoptosis, and inflammatory responses. Through complementary single-cell transcriptomic analysis and immunohistochemical validation, we mapped the distinct spatial distribution patterns of these markers. These findings identify a promising biomarker panel for the noninvasive diagnosis of DKD and provide important insights into tubular injury mechanisms. Moreover, the combined use of PDK4, RHCG, and FBP1 as a multi-compartment panel captures distinct yet complementary aspects of tubular injury. By integrating these signals, this strategy provides a more comprehensive assessment of disease pathology and represents a key step forward in the development of robust diagnostic signatures.

Current diagnosis of DKD relies on eGFR and proteinuria, but these markers show limited sensitivity in early stages. This underscores the need for better biomarkers. Urine provides a promising non-invasive source for such markers [30]. Studies like Ye et al. have used bulk transcriptome sequencing of urine to identify candidates such as PART1 and PLA2R1 [31]. However, this approach faces a critical limitation: urine contains mixed cell types, and bulk sequencing cannot separate signals from different cells [22]. Our data confirm this problem, showing that healthy urine contains abundant bladder and urethral cells, while DKD patients have significantly fewer. Such cellular differences mean that bulk transcriptome comparisons may yield misleading results. Although methods like those of Zhou et al. help confirm kidney specificity of markers like BBOX1, bulk analysis still cannot resolve cellular heterogeneity or link changes to specific kidney cell types [13]. This limitation restricts deeper mechanistic understanding of DKD.

In our current study, we overcame these limitations by innovatively integrating single-cell transcriptomic data from urine sediments with kidney tissue reference datasets. This approach enabled accurate differentiation between kidney-derived and non-kidney-derived cells at single-cell resolution. We successfully constructed a detailed cellular atlas comprising eight characteristic kidney cell subpopulations. This atlas not only reveals dynamic changes in key cellular populations during DKD progression but, more importantly, establishes a refined framework for developing cell type-specific diagnostic markers. The methodology also shows potential for application in other single-cell analysis contexts involving body fluids like cerebrospinal fluid, bronchoalveolar lavage fluid, and their corresponding tissues.

A key aspect of our study design was to identify diagnostic biomarkers by comparing urinary renal cells across DKD severity stages (early vs. advanced), rather than directly against healthy controls. This approach was driven by two key considerations. First, it was a matter of feasibility, as healthy control urine contained minimal renal cells for a valid comparison. Second, it was based on the biological rationale that genes central to disease progression are also likely to represent core diagnostic features. The success of this strategy is confirmed by the subsequent validation of the identified panel (FBP1, PDK4, RHCG) in independent kidney tissue cohorts, where it achieved excellent performance in distinguishing DKD from controls. Following this strategic discovery, we examined FBP1 and PDK4 expression across compartments. In urine, both genes were upregulated in Inj-PTC relative to prolif-PTC. In contrast, they were downregulated in Inj-PTC within kidney tissues compared to their normal counterparts. Importantly, further analysis revealed consistent downregulation of these biomarkers across multiple nephropathies beyond DKD, with RHCG demonstrating disease-specific expression patterns while FBP1 and PDK4 may reflect shared tubular injury responses. Bulk transcriptome analysis of DKD kidneys also showed significant downregulation of FBP1 and PDK4. These differential expression patterns between urinary and tissue-derived cells suggest that urine Inj-PTC and kidney tissue Inj-PTC subgroups may represent distinct biological states.

FBP1 (fructose-1,6-bisphosphatase 1) is a key rate-limiting enzyme in gluconeogenesis. It catalyzes the hydrolysis of fructose-1,6-bisphosphate to fructose-6-phosphate, a critical step for driving glucose production [32]. In the kidney, proximal tubule cells uniquely express gluconeogenic enzymes (e.g., FBP1, PCK1, G6PC) and rely on lactate as the primary substrate [33]. Impaired FBP1 activity disrupts glucose synthesis from non-carbohydrate precursors, reducing renal glucose output and lactate clearance [34]. Clinical evidence further supports its pathological relevance. In idiopathic nephrotic syndrome, urinary FBP1 activity is elevated during active proteinuria and normalizes following prednisone treatment. This dynamic pattern suggests that FBP1 serves as a biomarker of proximal tubule injury [35]. Notably, FBP1 expression is robust in healthy PTC but diminished in fibrotic regions. ScRNA-seq data reveals that proximal tubule specific gluconeogenic loss is an early hallmark of CKD, consistent across proteinuric, ischemic, and obstructive models [36]. Our data demonstrate significant FBP1 downregulation in Inj-PTC and across diverse CKD etiologies, indicating that reduced gluconeogenesis may be a common feature across different types of CKD. The co-downregulation of FBP1 and PDK4 points to a shared metabolic disruption. Measuring them together captures this synergy, creating a more powerful biomarker than any single one.

Pyruvate dehydrogenase kinase 4 (PDK4) is a mitochondrial matrix enzyme that promotes a metabolic shift from oxidative phosphorylation to glycolysis [37,38]. Notably, bioinformatics analyses and experimental evidence consistently demonstrate downregulated PDK4 expression in both membranous nephropathy patients and animal models [39], a phenomenon similarly observed in DKD [40]. This finding aligns with recent research showing that impaired glycolytic activity in peritubular endothelial cells contributes to microvascular rarefaction and fibrosis in CKD [41]. These observations collectively suggest that glycolytic dysfunction plays a pivotal role in microvascular injury. Our data further reveal a consistent reduction in PDK4 transcriptional levels across multiple CKD subtypes. In contrast to its downregulation in CKD, PDK4 is upregulated in acute kidney injury (AKI) models, where its deletion has been shown to ameliorate mitochondrial dysfunction and tubular damage [42,43]. This opposing expression suggests distinct, stage-specific metabolic adaptations in kidney disease, though current evidence remains largely correlative and derived from animal models.

Urine-derived renal cells represent a noninvasive source with significant clinical and research advantages over traditional tissue biopsies. Unlike invasive procedures, urine sampling is completely noninvasive, highly reproducible, and better accepted by patients, thereby enabling longitudinal monitoring of disease progression and therapeutic responses within the same individual [44]. Importantly, urine-derived cells not only maintain robust viability and proliferative capacity in vitro but also exhibit biological characteristics highly consistent with cells originating from renal tissue. These properties make them an ideal model for mechanistic studies and therapeutic evaluation in kidney diseases [10].

While this study provides novel insights into urinary single-cell biomarkers for DKD, several limitations should be acknowledged. First, First, our analysis relied exclusively on public, retrospective datasets. Despite integrating multiple cohorts and applying batch-effect correction, our findings are limited by the relatively small subject numbers and the potential for residual batch effects across different GEO platforms. Crucially, demonstrating these genes’ presence in urinary sediments does not equate to a clinically applicable assay. Future studies must employ targeted techniques (e.g., qPCR or protein assays) in independent, prospective cohorts to validate the diagnostic performance of this three-gene panel. Furthermore, such studies are needed to establish its clinical utility as a robust liquid biopsy for DKD, which includes direct comparisons against other CKD etiologies to confirm its specificity.

Second, although our bioinformatics approach rigorously distinguished renal-origin cells from contaminants, further experimental validation is required to confirm the molecular mechanisms underlying tubular injury and the specific role of FBP1/PDK4 dysregulation in proximal tubule shedding. Additionally, the potential confounding effects of clinical variables such as age, sex, diabetes duration, and baseline eGFR could not be fully addressed due to data constraints. Finally, the modest cohort sizes present a risk of overfitting. Consequently, larger prospective multicenter studies are warranted to robustly validate this signature’s clinical applicability.

## Conclusion

By integrating urinary and renal single-cell sequencing with machine learning, this study identified a tubular injury-associated gene signature (FBP1, PDK4, and RHCG) that is detectable in urinary cells and shows a strong association with DKD. This signature represents a promising candidate for the development of a non-invasive diagnostic assay. These findings not only deepen our understanding of tubular injury mechanisms in DKD but also advance the transition of renal disease diagnostics from conventional markers to precision medicine. Future large-scale, longitudinal validation studies are warranted to confirm its clinical utility and facilitate development of standardized urine-based diagnostic assays for DKD.

## Supporting information

S1 File Supplementary methods. (DOCX)

S1 Fig Dataset integration and gene signature pipeline for DKD. (DOCX)

S2 Fig Analysis of macrophage dynamics and cell communication in urinary sediments. (DOCX)

S3 Fig Additional validation of diagnostic models and biomarker expression. (DOCX)

S4 Fig Expression of diagnostic biomarkers across various kidney diseases. (DOCX)

S5 Fig Comparison of gene expression profiles between diabetic kidney disease and other chronic kidney diseases. (DOCX)

S1 Table Diagnostic performance of individual candidate genes identified by LASSO regression. (DOCX)

S2 Table Performance of multivariate diagnostic models built with the three-gene panel (PDK4, RHCG, FBP1). (DOCX)

## Abbreviations

DKD: diabetic kidney disease
CKD: chronic kidney disease
ESRD: end-stage renal disease
eGFR: estimated glomerular filtration rate
GSVA: gene set variation analysis
GEO: Gene Expression Omnibus
KEGG: Kyoto Encyclopedia of Genes and Genomes
LASSO: least absolute shrinkage and selection operator
DEGs: Differentially expressed genes
FDR: false discovery rate
ROC: receiver operating characteristic
LR: logistic regression
XGBoost: eXtreme Gradient Boosting
SVM: support vector machines
RF: random forest
AUC: area under the curve
PPI: protein-protein interaction
CD-PC: collecting duct principal cell
PTC: proximal tubule cells
DCT: distal convoluted tubule
Inj-PTC: damaged PTC
Imm: immune
Mes: mesangial
Podo: podocytes
EC: endothelial cell
CD-ICA: collecting duct intercalated type-A
CD-ICB: collecting duct intercalated type-B
LOH: loop of Henle
Inj-LOH: injured loop of Henle
Prolif-PTC: Proliferative PTC
Inf-Mac: inflammatory macrophages
MCD: Minimal change disease
LN: lupus nephritis
FSG: focal segmental glomerulosclerosis
IgAN: IgA nephropathy
MGN: membranous glomerulonephritis

## References

[1] Tuttle KR, Wong L, St Peter W, Roberts G, Rangaswami J, Mottl A, et al. Moving from evidence to implementation of breakthrough therapies for diabetic kidney disease. Clin J Am Soc Nephrol. 2022;17(7):1092–103. doi: 10.2215/CJN.02980322 35649722 PMC9269635

[2] Foreman KJ, Marquez N, Dolgert A, Fukutaki K, Fullman N, McGaughey M, et al. Forecasting life expectancy, years of life lost, and all-cause and cause-specific mortality for 250 causes of death: reference and alternative scenarios for 2016-40 for 195 countries and territories. Lancet. 2018;392(10159):2052–90. doi: 10.1016/S0140-6736(18)31694-5 30340847 PMC6227505

[3] Wang Y, Lin T, Lu J, He W, Chen H, Wen T, et al. Trends and analysis of risk factor differences in the global burden of chronic kidney disease due to type 2 diabetes from 1990 to 2021: a population-based study. Diabetes Obes Metab. 2025;27(4):1902–19. doi: 10.1111/dom.16183 39806549

[4] Wang Y, Gu S, Xie Z, Xu Z, He W, Chen Y, et al. Trends and disparities in the burden of chronic kidney disease due to type 2 diabetes in China from 1990 to 2021: a population-based study. J Diabetes. 2025;17(4):e70084. doi: 10.1111/1753-0407.70084 40265496 PMC12015641

[5] Tumlin JA, Campbell KN. Proteinuria in nephrotic syndrome: mechanistic and clinical considerations in optimizing management. Am J Nephrol. 2018;47 Suppl 1:1–2. doi: 10.1159/000481632 29852500

[6] Jung C-Y, Yoo T-H. Pathophysiologic mechanisms and potential biomarkers in diabetic kidney disease. Diabetes Metab J. 2022;46(2):181–97. doi: 10.4093/dmj.2021.0329 35385633 PMC8987689

[7] Poggio ED, McClelland RL, Blank KN, Hansen S, Bansal S, Bomback AS, et al. Systematic review and meta-analysis of native kidney biopsy complications. Clin J Am Soc Nephrol. 2020;15(11):1595–602. doi: 10.2215/CJN.04710420 33060160 PMC7646247

[8] Nikanjam M, Kato S, Kurzrock R. Liquid biopsy: current technology and clinical applications. J Hematol Oncol. 2022;15(1):131. doi: 10.1186/s13045-022-01351-y 36096847 PMC9465933

[9] Wani ZA, Ahmed S, Saleh A, Anna VR, Fahelelbom KM, Raju SK, et al. Biomarkers in diabetic nephropathy: a comprehensive review of their role in early detection and disease progression monitoring. Diabetes Res Clin Pract. 2025;226:112292. doi: 10.1016/j.diabres.2025.112292 40466742

[10] Soltani-Fard E, Taghvimi S, Karimi F, Vahedi F, Khatami SH, Behrooj H, et al. Urinary biomarkers in diabetic nephropathy. Clin Chim Acta. 2024;561:119762. doi: 10.1016/j.cca.2024.119762 38844018

[11] de Carvalho JAM, Tatsch E, Hausen BS, Bollick YS, Moretto MB, Duarte T, et al. Urinary kidney injury molecule-1 and neutrophil gelatinase-associated lipocalin as indicators of tubular damage in normoalbuminuric patients with type 2 diabetes. Clin Biochem. 2016;49(3):232–6. doi: 10.1016/j.clinbiochem.2015.10.016 26519090

[12] Feng S-T, Yang Y, Yang J-F, Gao Y-M, Cao J-Y, Li Z-L, et al. Urinary sediment CCL5 messenger RNA as a potential prognostic biomarker of diabetic nephropathy. Clin Kidney J. 2021;15(3):534–44. doi: 10.1093/ckj/sfab186 35211307 PMC8862108

[13] Zhou L-T, Lv L-L, Qiu S, Yin Q, Li Z-L, Tang T-T, et al. Bioinformatics-based discovery of the urinary BBOX1 mRNA as a potential biomarker of diabetic kidney disease. J Transl Med. 2019;17(1):59. doi: 10.1186/s12967-019-1818-2 30819181 PMC6394064

[14] Abedini A, Zhu YO, Chatterjee S, Halasz G, Devalaraja-Narashimha K, Shrestha R, et al. Urinary single-cell profiling captures the cellular diversity of the kidney. J Am Soc Nephrol. 2021;32(3):614–27. doi: 10.1681/ASN.2020050757 33531352 PMC7920183

[15] Klocke J, Kim SJ, Skopnik CM, Hinze C, Boltengagen A, Metzke D, et al. Urinary single-cell sequencing captures kidney injury and repair processes in human acute kidney injury. Kidney Int. 2022;102(6):1359–70. doi: 10.1016/j.kint.2022.07.032 36049643

[16] Satija R, Farrell JA, Gennert D, Schier AF, Regev A. Spatial reconstruction of single-cell gene expression data. Nat Biotechnol. 2015;33(5):495–502. doi: 10.1038/nbt.3192 25867923 PMC4430369

[17] Butler A, Hoffman P, Smibert P, Papalexi E, Satija R. Integrating single-cell transcriptomic data across different conditions, technologies, and species. Nat Biotechnol. 2018;36(5):411–20. doi: 10.1038/nbt.4096 29608179 PMC6700744

[18] Stuart T, Butler A, Hoffman P, Hafemeister C, Papalexi E, Mauck WM 3rd, et al. Comprehensive integration of single-cell data. Cell. 2019;177(7):1888-1902.e21. doi: 10.1016/j.cell.2019.05.031 31178118 PMC6687398

[19] Hao Y, Hao S, Andersen-Nissen E, Mauck WM 3rd, Zheng S, Butler A, et al. Integrated analysis of multimodal single-cell data. Cell. 2021;184(13):3573-3587.e29. doi: 10.1016/j.cell.2021.04.048 34062119 PMC8238499

[20] Yu Z, Liao J, Chen Y, Zou C, Zhang H, Cheng J, et al. Single-cell transcriptomic map of the human and mouse bladders. J Am Soc Nephrol. 2019;30(11):2159–76. doi: 10.1681/ASN.2019040335 31462402 PMC6830796

[21] Gouin KH 3rd, Ing N, Plummer JT, Rosser CJ, Ben Cheikh B, Oh C, et al. An N-Cadherin 2 expressing epithelial cell subpopulation predicts response to surgery, chemotherapy and immunotherapy in bladder cancer. Nat Commun. 2021;12(1):4906. doi: 10.1038/s41467-021-25103-7 34385456 PMC8361097

[22] Ma D, Wang D, Yu J, Huang N, Luo N, Yang Y, et al. Single-cell profiling of tubular epithelial cells in adaptive state in the urine sediment of patients with early and advanced diabetic kidney disease. Kidney Int Rep. 2024;10(3):892–905. doi: 10.1016/j.ekir.2024.11.002 40225386 PMC11993230

[23] Hu C, Li T, Xu Y, Zhang X, Li F, Bai J, et al. CellMarker 2.0: an updated database of manually curated cell markers in human/mouse and web tools based on scRNA-seq data. Nucleic Acids Res. 2023;51(D1):D870–6. doi: 10.1093/nar/gkac947 36300619 PMC9825416

[24] Qiu X, Mao Q, Tang Y, Wang L, Chawla R, Pliner HA, et al. Reversed graph embedding resolves complex single-cell trajectories. Nat Methods. 2017;14(10):979–82. doi: 10.1038/nmeth.4402 28825705 PMC5764547

[25] Qiu X, Hill A, Packer J, Lin D, Ma Y-A, Trapnell C. Single-cell mRNA quantification and differential analysis with Census. Nat Methods. 2017;14(3):309–15. doi: 10.1038/nmeth.4150 28114287 PMC5330805

[26] Trapnell C, Cacchiarelli D, Grimsby J, Pokharel P, Li S, Morse M, et al. The dynamics and regulators of cell fate decisions are revealed by pseudotemporal ordering of single cells. Nat Biotechnol. 2014;32(4):381–6. doi: 10.1038/nbt.2859 24658644 PMC4122333

[27] Jin S, Guerrero-Juarez CF, Zhang L, Chang I, Ramos R, Kuan C-H, et al. Inference and analysis of cell-cell communication using CellChat. Nat Commun. 2021;12(1):1088. doi: 10.1038/s41467-021-21246-9 33597522 PMC7889871

[28] Liberzon A, Birger C, Thorvaldsdóttir H, Ghandi M, Mesirov JP, Tamayo P. The Molecular Signatures Database (MSigDB) hallmark gene set collection. Cell Syst. 2015;1(6):417–25. doi: 10.1016/j.cels.2015.12.004 26771021 PMC4707969

[29] Fitzgerald M, Saville BR, Lewis RJ. Decision curve analysis. JAMA. 2015;313(4):409–10. doi: 10.1001/jama.2015.37 25626037

[30] Khan NU, Lin J, Liu X, Li H, Lu W, Zhong Z, et al. Insights into predicting diabetic nephropathy using urinary biomarkers. Biochim Biophys Acta Proteins Proteom. 2020;1868(10):140475. doi: 10.1016/j.bbapap.2020.140475 32574766

[31] Ye Q, Xu G, Yuan H, Mi J, Xie Y, Li H, et al. Urinary PART1 and PLA2R1 could potentially serve as diagnostic markers for diabetic kidney disease patients. Diabetes Metab Syndr Obes. 2023;16:4215–31. doi: 10.2147/DMSO.S445341 38162802 PMC10757812

[32] Bertinat R, Pontigo JP, Pérez M, Concha II, San Martín R, Guinovart JJ, et al. Nuclear accumulation of fructose 1,6-bisphosphatase is impaired in diabetic rat liver. J Cell Biochem. 2012;113(3):848–56. doi: 10.1002/jcb.23413 22021109

[33] Mather A, Pollock C. Glucose handling by the kidney. Kidney Int Suppl. 2011;(120):S1-6. doi: 10.1038/ki.2010.509 21358696

[34] Legouis D, Ricksten S-E, Faivre A, Verissimo T, Gariani K, Verney C, et al. Altered proximal tubular cell glucose metabolism during acute kidney injury is associated with mortality. Nat Metab. 2020;2(8):732–43. doi: 10.1038/s42255-020-0238-1 32694833

[35] Kepka A, Dariusz Szajda S, Stypułkowska A, Waszkiewicz N, Jankowska A, Chojnowska S, et al. Urinary fructose-1,6-bisphosphatase activity as a marker of the damage to the renal proximal tubules in children with idiopathic nephrotic syndrome. Clin Chem Lab Med. 2008;46(6):831–5. doi: 10.1515/CCLM.2008.171 18601606

[36] Verissimo T, Faivre A, Rinaldi A, Lindenmeyer M, Delitsikou V, Veyrat-Durebex C, et al. Decreased renal gluconeogenesis is a hallmark of chronic kidney disease. J Am Soc Nephrol. 2022;33(4):810–27. doi: 10.1681/ASN.2021050680 35273087 PMC8970457

[37] Lee I-K. The role of pyruvate dehydrogenase kinase in diabetes and obesity. Diabetes Metab J. 2014;38(3):181–6. doi: 10.4093/dmj.2014.38.3.181 25003070 PMC4083023

[38] Thapa D, Stoner MW, Zhang M, Xie B, Manning JR, Guimaraes D, et al. Adropin regulates pyruvate dehydrogenase in cardiac cells via a novel GPCR-MAPK-PDK4 signaling pathway. Redox Biol. 2018;18:25–32. doi: 10.1016/j.redox.2018.06.003 29909017 PMC6008287

[39] Han M, Wang Y, Huang X, Li P, Shan W, Gu H, et al. Prediction of biomarkers associated with membranous nephropathy: bioinformatic analysis and experimental validation. Int Immunopharmacol. 2024;126:111266. doi: 10.1016/j.intimp.2023.111266 38029552

[40] Han Y, Jin L, Wang L, Wei L, Tu C. Identification of PDK4 as hub gene for diabetic nephropathy using co-expression network analysis. Kidney Blood Press Res. 2023;48(1):522–34. doi: 10.1159/000531288 37385224 PMC10619590

[41] Huang Y, Cong A, Li J, Zhou Z, Zhou H, Su C, et al. Glycolysis in peritubular endothelial cells and microvascular rarefaction in CKD. J Am Soc Nephrol. 2025;36(1):19–33. doi: 10.1681/ASN.0000000000000488 39226371 PMC11706556

[42] Oh CJ, Ha C-M, Choi Y-K, Park S, Choe MS, Jeoung NH, et al. Pyruvate dehydrogenase kinase 4 deficiency attenuates cisplatin-induced acute kidney injury. Kidney Int. 2017;91(4):880–95. doi: 10.1016/j.kint.2016.10.011 28040265

[43] Oh CJ, Kim M-J, Lee J-M, Kim DH, Kim I-Y, Park S, et al. Inhibition of pyruvate dehydrogenase kinase 4 ameliorates kidney ischemia-reperfusion injury by reducing succinate accumulation during ischemia and preserving mitochondrial function during reperfusion. Kidney Int. 2023;104(4):724–39. doi: 10.1016/j.kint.2023.06.022 37399974

[44] Oliveira Arcolino F, Tort Piella A, Papadimitriou E, Bussolati B, Antonie DJ, Murray P, et al. Human urine as a noninvasive source of kidney cells. Stem Cells Int. 2015;2015:362562. doi: 10.1155/2015/362562 26089913 PMC4451513

