# Supplementary material for: A non-invasive urinary diagnostic signature for diabetic kidney disease revealed by machine learning and single-cell analysis
Source: PLoS One. 2026 Jan 2;21(1):e0340096. doi: 10.1371/journal.pone.0340096 (PMC12758759; doi:10.1371/journal.pone.0340096)
Supplement: S1 File — (DOCX) [file pone.0340096.s001.docx]

**Supplementary Methods**

**Quality Control, Integration, and Clustering of Single-Cell RNA-Sequencing Data**

Single-cell RNA sequencing data from DKD renal tissues and urinary sediments were processed using Seurat (v5.3.0). For renal tissue data (GSE131882), stringent quality control was applied using the following criteria to exclude low-quality cells: cells with fewer than three detected genes, a unique gene count (nFeature_RNA) of less than 300 or greater than 5,000, a total UMI count (nCount_RNA) exceeding 20,000, or a mitochondrial gene percentage (percent.mt) above 15%. This filtering process resulted in the retention of 20,220 high-quality renal cells from an initial pool of 23,980 for downstream analysis.

For urinary sediment data (sourced from GSE266146 and GSE157640), quality control was performed with adjusted thresholds (nFeature_RNA < 250; nCount_RNA < 500; percent.mt > 15%) to account for the lower RNA content and higher debris contamination typical of urinary samples. Application of these criteria resulted in a final set of 3,421 high-quality urinary cells from an initial 5,551.

Following quality control, data integration to address batch effects was performed using Harmony (v1.2.3). Expression data were normalized via the LogNormalize method (scale factor = 10,000) and scaled through z-score transformation based on 2,000 highly variable genes identified by mean-variance modeling. Dimensional reduction was conducted using the top 50 principal components. Cell clustering was performed on the integrated data using the Harmony reduction in the FindClusters function. To obtain biologically relevant clusters, resolution was set to 0.5 for renal tissue data and 0.4 for urinary sediment data. Cell types were annotated based on the expression of canonical markers established in published renal single-cell studies[1, 2].

To systematically discriminate bladder/urethral epithelial contaminants from authentic renal cells in urine sediments, we implemented an anchor-based integration strategy[3]. Following independent LogNormalize normalization and variable feature selection (2,000 genes) while preserving bladder and urethra specific markers (PSCA, PLAT, KRT13, FXYD4)[4, 5], cross-tissue integration was performed using RPCA-based dimensional reduction (dims=1:30, k.anchor=20) and subsequent data fusion. The integrated dataset underwent joint dimensional reduction (50 principal components) and UMAP visualization (dims=1:30). Contaminant identification employed stringent dual criteria based on both spatial segregation patterns and marker gene expression—where putative contaminants formed distinct clusters separate from renal populations in UMAP space while simultaneously exhibiting elevated expression of multiple bladder/urethral epithelial markers, with expression levels systematically exceeding those observed in renal cell populations. This dual-criteria approach specifically targeted non-renal cells while maintaining renal population integrity, thereby extending conventional integration methodologies through biologically-informed computational filtering.

Reference

1. Wilson, P.C., H. Wu, Y. Kirita, K. Uchimura, N. Ledru, H.G. Rennke, et al., *The single-cell transcriptomic landscape of early human diabetic nephropathy.* Proc Natl Acad Sci U S A, 2019. **116**(39): p. 19619-19625.

2. Abedini, A., Y.O. Zhu, S. Chatterjee, G. Halasz, K. Devalaraja-Narashimha, R. Shrestha, et al., *Urinary Single-Cell Profiling Captures the Cellular Diversity of the Kidney.* J Am Soc Nephrol, 2021. **32**(3): p. 614-627.

3. Stuart, T., A. Butler, P. Hoffman, C. Hafemeister, E. Papalexi, W.M. Mauck, 3rd, et al., *Comprehensive Integration of Single-Cell Data.* Cell, 2019. **177**(7): p. 1888-1902.e21.

4. Yu, Z., J. Liao, Y. Chen, C. Zou, H. Zhang, J. Cheng, et al., *Single-Cell Transcriptomic Map of the Human and Mouse Bladders.* J Am Soc Nephrol, 2019. **30**(11): p. 2159-2176.

5. Gouin, K.H., 3rd, N. Ing, J.T. Plummer, C.J. Rosser, B. Ben Cheikh, C. Oh, et al., *An N-Cadherin 2 expressing epithelial cell subpopulation predicts response to surgery, chemotherapy and immunotherapy in bladder cancer.* Nat Commun, 2021. **12**(1): p. 4906.
