# Supplementary material for: A non-invasive urinary diagnostic signature for diabetic kidney disease revealed by machine learning and single-cell analysis
Source: PLoS One. 2026 Jan 2;21(1):e0340096. doi: 10.1371/journal.pone.0340096 (PMC12758759; doi:10.1371/journal.pone.0340096)
Supplement: S1 Fig — (DOCX) [file pone.0340096.s002.docx]

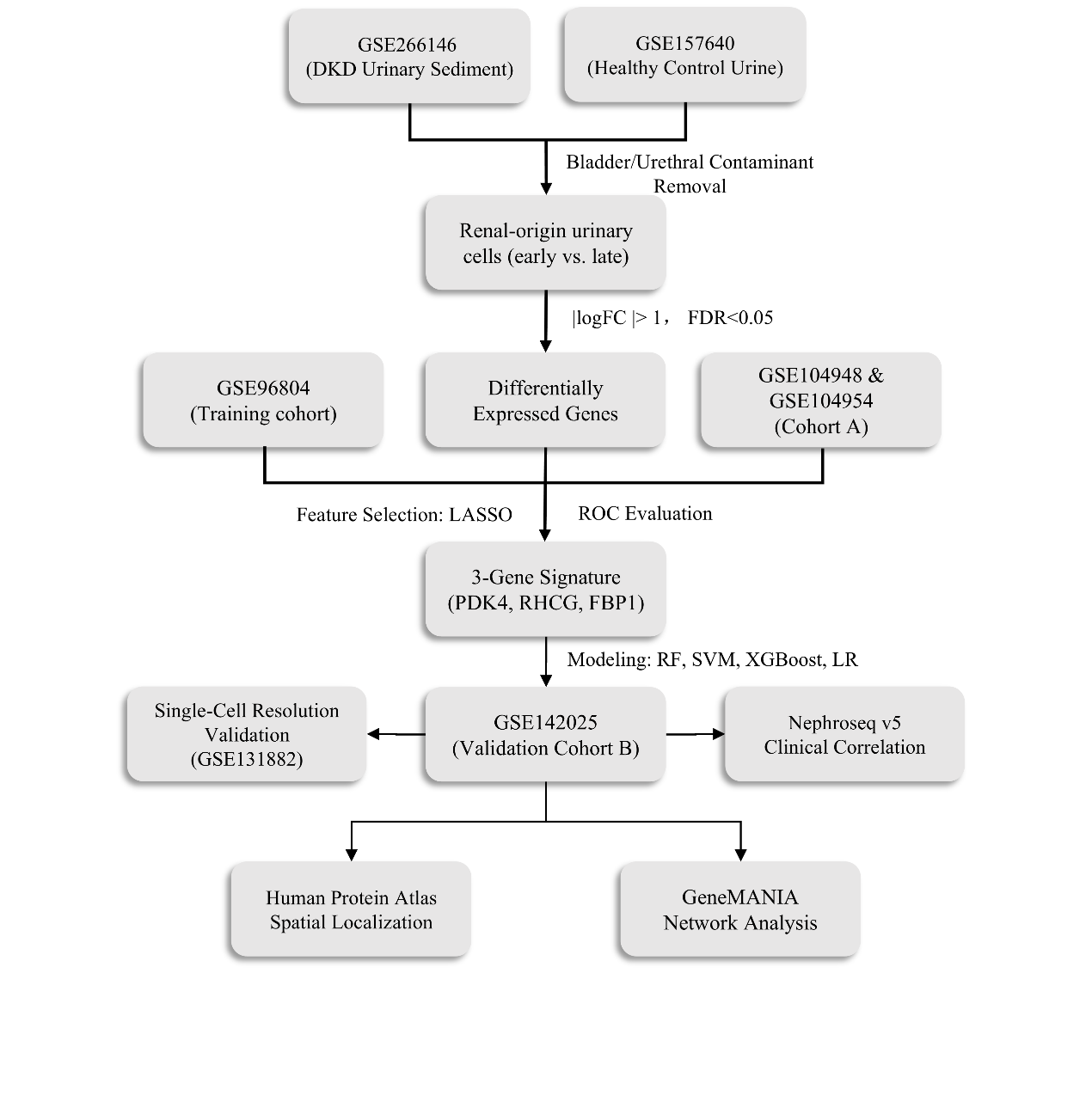


**S1 Fig: Dataset Integration and Gene Signature Pipeline for DKD.**

Abbreviations: DKD, diabetic kidney disease; FDR, false discovery rate; LASSO, Least Absolute Shrinkage and Selection Operator; ROC, Receiver Operating Characteristic; RF, random forest; SVM, support vector machines; XGBoost, eXtreme Gradient Boosting; LR, logistic regression.
