# Supplementary material for: A non-invasive urinary diagnostic signature for diabetic kidney disease revealed by machine learning and single-cell analysis
Source: PLoS One. 2026 Jan 2;21(1):e0340096. doi: 10.1371/journal.pone.0340096 (PMC12758759; doi:10.1371/journal.pone.0340096)
Supplement: S2 Fig — (DOCX) [file pone.0340096.s003.docx]

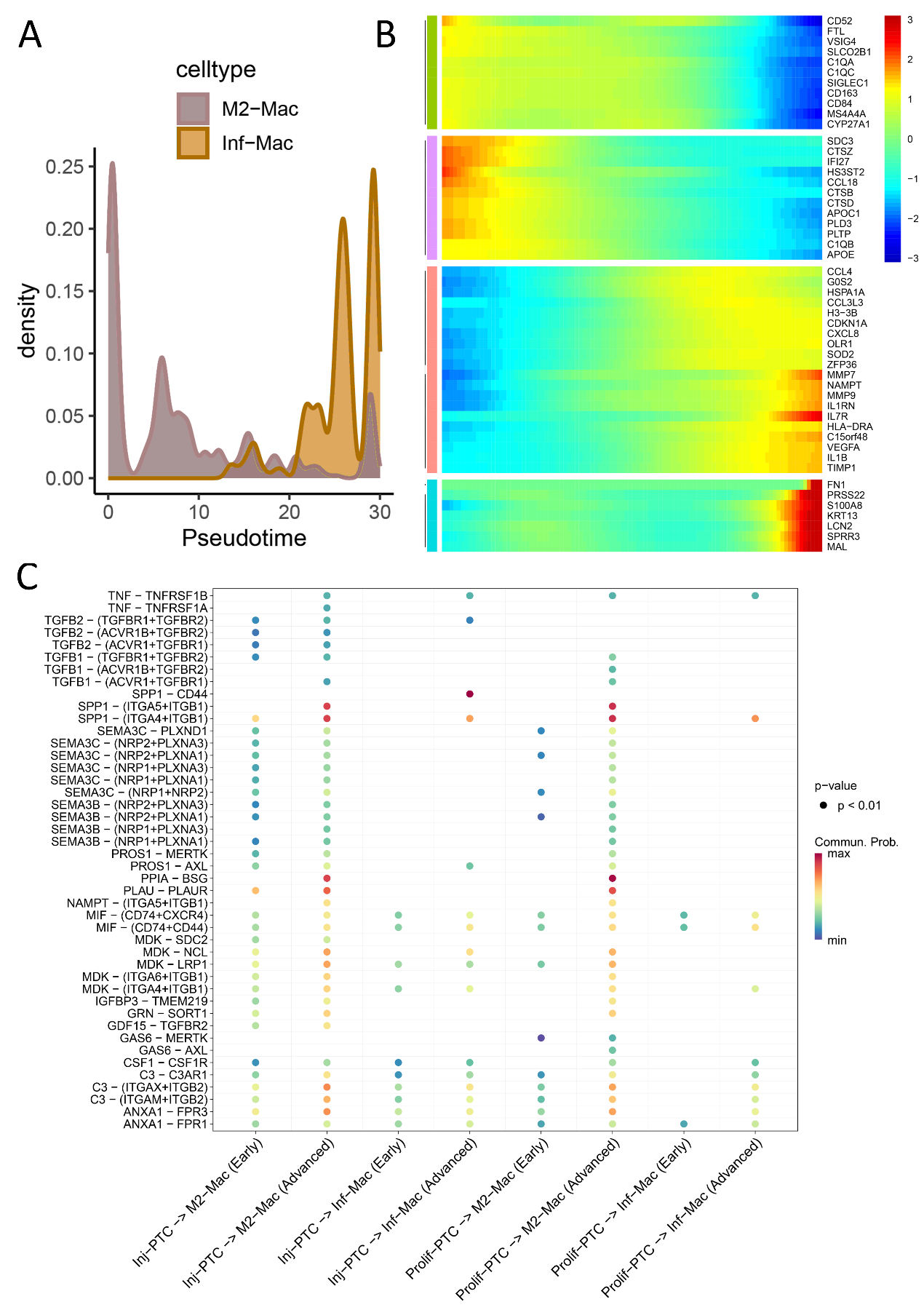


**S2 Fig. Analysis of macrophage dynamics and cell communication in urinary sediments.**

(A-B) Pseudotime trajectory analysis of macrophage subpopulations. (A) Cell distribution along the inferred state transition. Cells are ordered by pseudotime, representing a continuum from M2-like macrophages (M2-Mac, purple) to inflammatory macrophages (Inf-Mac, gray). (B) Dynamic gene expression changes. The heatmap shows expression patterns for key genes across the pseudotime trajectory (columns: cells; rows: genes). The color scale (blue to red) indicates low to high expression levels, highlighting genes activated or suppressed during the macrophage transition. (C) CellChat analysis bubble plot depicting significant ligand-receptor interactions between PTC and macrophage subpopulations. The color and size of the bubbles represent the communication probability.
