# Supplementary material for: A non-invasive urinary diagnostic signature for diabetic kidney disease revealed by machine learning and single-cell analysis
Source: PLoS One. 2026 Jan 2;21(1):e0340096. doi: 10.1371/journal.pone.0340096 (PMC12758759; doi:10.1371/journal.pone.0340096)
Supplement: S3 Fig — (DOCX) [file pone.0340096.s004.docx]

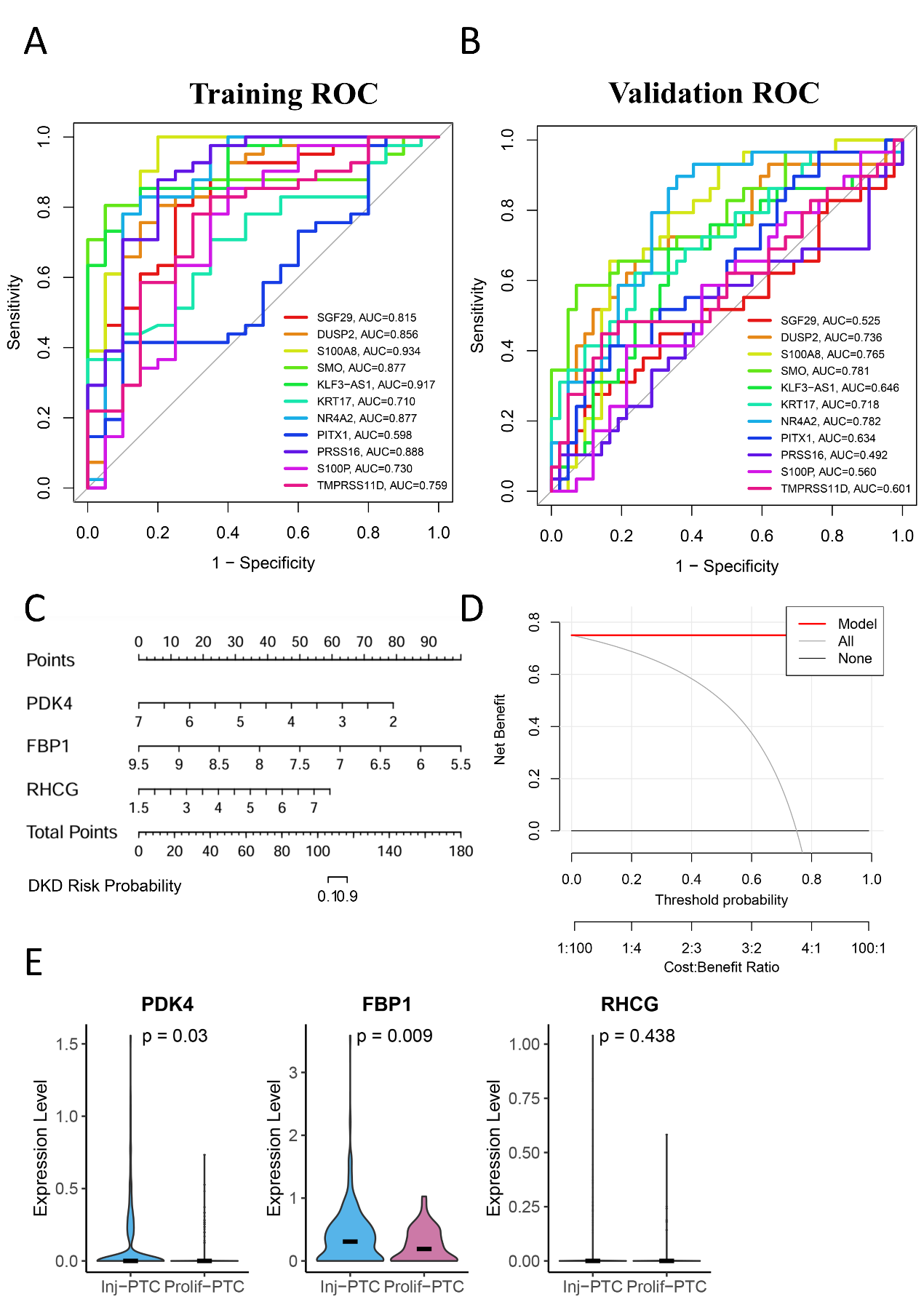


**S3 Fig. Additional validation of diagnostic models and biomarker expression.**

(A-B) ROC curves of the remaining 11 candidate genes in the GSE96804 training set (n=40 DKD vs 21 controls) (A) and GSE104948/54 validation sets (n=30 DKD vs 42 controls) (B). (C-D) Clinical utility assessment of the multi-gene model. (C) Nomogram for predicting DKD probability based on the three-gene signature. (D) Decision curve analysis (DCA) comparing the net benefit of the model against default strategies. (E) Violin plots showing the expression of PDK4, RHCG, and FBP1 in Inj-PTC (n=385) versus prolif-PTC (n=98) clusters within urinary sediments. Differential expression for each gene was performed using the Wilcoxon rank-sum test; p-values are unadjusted. The black horizontal bar represents the median expression value.
