# Supplementary material for: A non-invasive urinary diagnostic signature for diabetic kidney disease revealed by machine learning and single-cell analysis
Source: PLoS One. 2026 Jan 2;21(1):e0340096. doi: 10.1371/journal.pone.0340096 (PMC12758759; doi:10.1371/journal.pone.0340096)
Supplement: S4 Fig — (DOCX) [file pone.0340096.s005.docx]

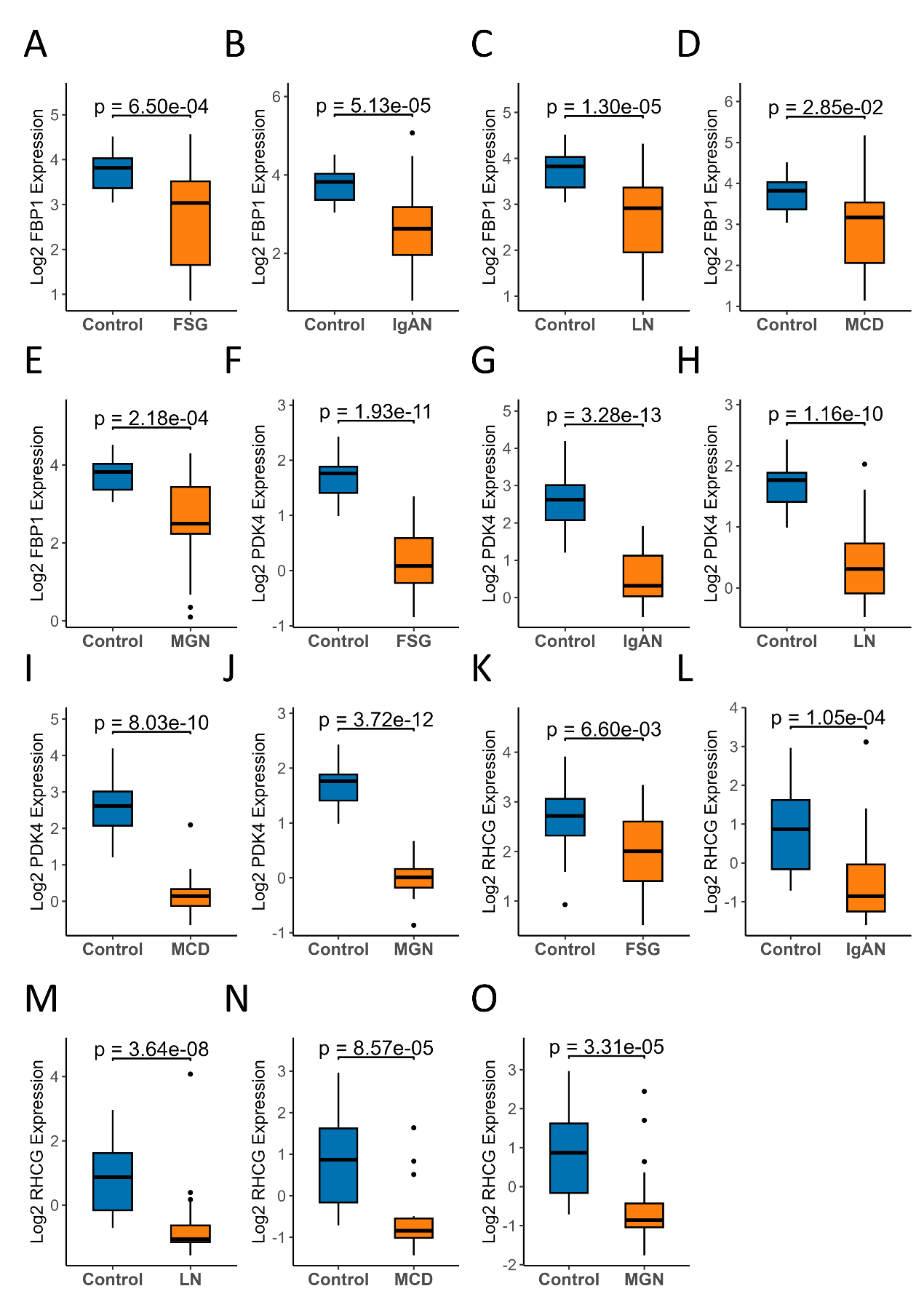


**S4 Fig. Expression of diagnostic biomarkers across various kidney diseases.**

Box plots showing the expression levels of PDK4, RHCG, and FBP1 (presented as Z-scores) in bulk tissue transcriptomes from the Nephroseq database, comparing controls with patients with DKD, minimal change disease (MCD), lupus nephritis (LN), focal segmental glomerulosclerosis (FSGS), IgA nephropathy (IgAN), and membranous glomerulonephritis (MGN). Each disease cohort was compared to the healthy control group using Wilcoxon rank-sum tests. Resulting p-values are unadjusted. Sample sizes: Control n=21, DKD n=12, MCD n=14, LN n=31, FSGS n=25, IgAN n=27, MGN n=21.
