# Supplementary material for: A non-invasive urinary diagnostic signature for diabetic kidney disease revealed by machine learning and single-cell analysis
Source: PLoS One. 2026 Jan 2;21(1):e0340096. doi: 10.1371/journal.pone.0340096 (PMC12758759; doi:10.1371/journal.pone.0340096)
Supplement: S5 Fig — (DOCX) [file pone.0340096.s006.docx]

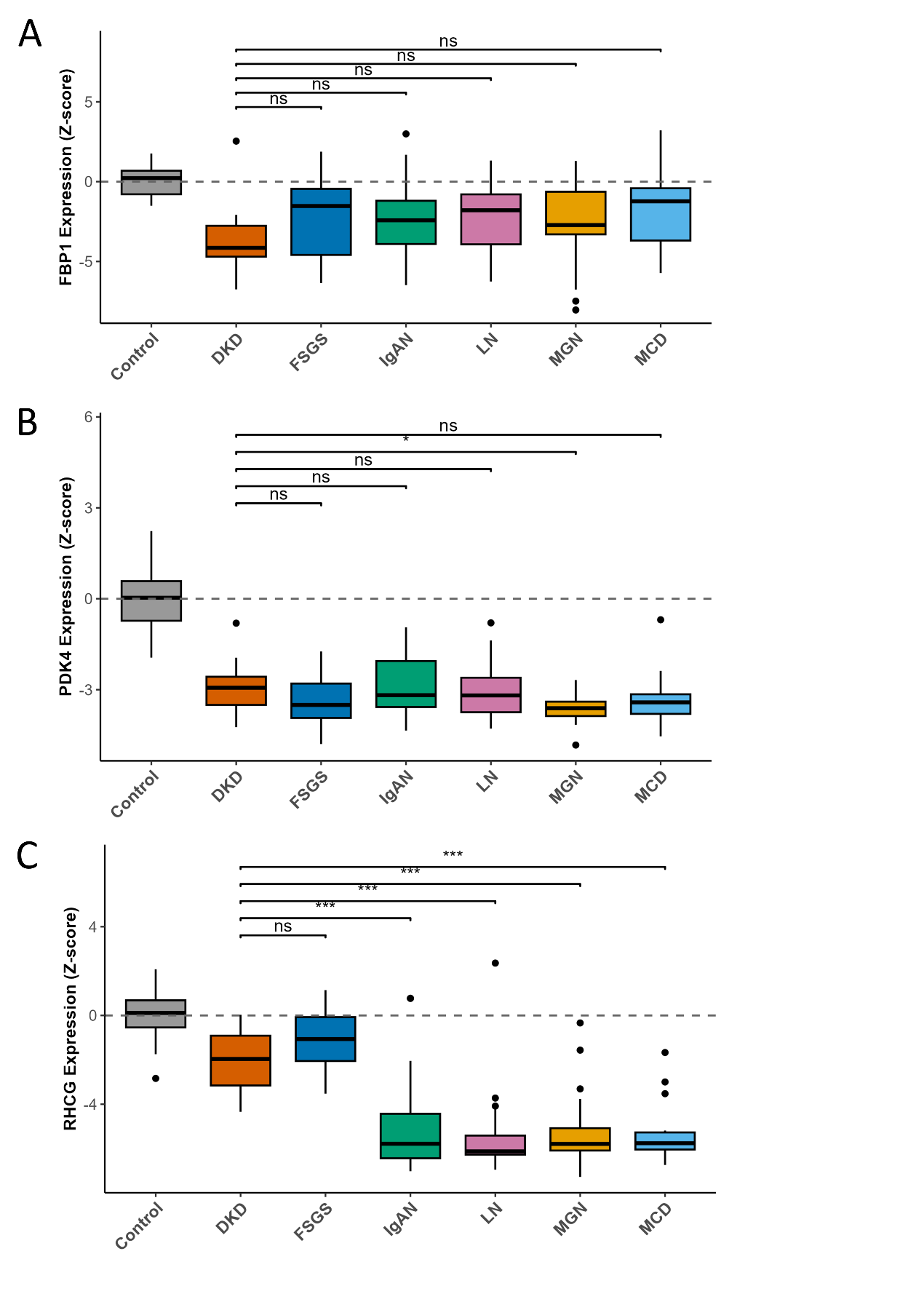


**S5 Fig. Comparison of gene expression profiles between diabetic kidney disease and other chronic kidney diseases.**

**Box plots show expression levels of (A) FBP1, (B) PDK4, and (C) RHCG across different kidney disease types. Data are presented as Z-scores normalized relative to healthy controls (n=21). Five pre-planned comparisons between DKD and each other CKD type (FSGS, IgAN, LN, MGN, MCD) were performed for each gene using two-sided Mann-Whitney U tests with Bonferroni correction for multiple testing. Sample sizes: DKD n=12, FSGS n=25, IgAN n=27, LN n=31, MGN n=21, MCD n=14. *p < 0.05, **p < 0.01, ***p < 0.001; ns, not significant.**
