# Supplementary material for: A non-invasive urinary diagnostic signature for diabetic kidney disease revealed by machine learning and single-cell analysis
Source: PLoS One. 2026 Jan 2;21(1):e0340096. doi: 10.1371/journal.pone.0340096 (PMC12758759; doi:10.1371/journal.pone.0340096)
Supplement: S1 Table — (DOCX) [file pone.0340096.s007.docx]

**S1 Table. Diagnostic performance of individual candidate genes identified by LASSO regression.**

| Gene Symbol | Cohort | AUC | 95% CI | Sensitivity | Specificity |
| --- | --- | --- | --- | --- | --- |
|  |  |  |  |  |  |
| DUSP2 | Training (GSE96804) | 0.856 | 0.733-0.950 | 0.756 | 0.850 |
| DUSP2 | Validation A (GSE104948/54) | 0.736 | 0.605-0.851 | 0.690 | 0.738 |
| **FBP1** | Training (GSE96804) | 0.879 | 0.783-0.957 | 0.756 | 0.900 |
| **FBP1** | Validation A (GSE104948/54) | 0.853 | 0.756-0.939 | 0.586 | 1.000 |
| KLF3-AS1 | Training (GSE96804) | 0.917 | 0.840-0.974 | 0.854 | 0.850 |
| KLF3-AS1 | Validation A (GSE104948/54) | 0.646 | 0.515-0.779 | 0.690 | 0.667 |
| KRT17 | Training (GSE96804) | 0.710 | 0.579-0.841 | 0.366 | 1.000 |
| KRT17 | Validation A (GSE104948/54) | 0.718 | 0.591-0.836 | 0.621 | 0.762 |
| NR4A2 | Training (GSE96804) | 0.877 | 0.754-0.971 | 0.780 | 0.900 |
| NR4A2 | Validation A (GSE104948/54) | 0.782 | 0.667-0.882 | 0.897 | 0.643 |
| **PDK4** | Training (GSE96804) | 0.990 | 0.971-1.000 | 0.976 | 0.950 |
| **PDK4** | Validation A (GSE104948/54) | 0.986 | 0.953-1.000 | 0.966 | 0.976 |
| PITX1 | Training (GSE96804) | 0.598 | 0.443-0.743 | 0.415 | 0.900 |
| PITX1 | Validation A (GSE104948/54) | 0.634 | 0.496-0.762 | 0.414 | 0.833 |
| PRSS16 | Training (GSE96804) | 0.888 | 0.784-0.968 | 0.878 | 0.800 |
| PRSS16 | Validation A (GSE104948/54) | 0.492 | 0.356-0.636 | 0.552 | 0.571 |
| **RHCG** | Training (GSE96804) | 0.821 | 0.700-0.918 | 0.756 | 0.850 |
| **RHCG** | Validation A (GSE104948/54) | 0.834 | 0.722-0.923 | 0.862 | 0.690 |
| S100A8 | Training (GSE96804) | 0.934 | 0.851-0.994 | 1.000 | 0.800 |
| S100A8 | Validation A (GSE104948/54) | 0.765 | 0.645-0.872 | 0.655 | 0.833 |
| S100P | Training (GSE96804) | 0.730 | 0.577-0.872 | 0.854 | 0.600 |
| S100P | Validation A (GSE104948/54) | 0.560 | 0.426-0.684 | 0.414 | 0.786 |
| SGF29 | Training (GSE96804) | 0.815 | 0.683-0.921 | 0.805 | 0.750 |
| SGF29 | Validation A (GSE104948/54) | 0.525 | 0.385-0.672 | 0.276 | 0.881 |
| SMO | Training (GSE96804) | 0.877 | 0.784-0.956 | 0.805 | 0.950 |
| SMO | Validation A (GSE104948/54) | 0.781 | 0.654-0.883 | 0.586 | 0.929 |
| TMPRSS11D | Training (GSE96804) | 0.759 | 0.627-0.883 | 0.780 | 0.700 |
| TMPRSS11D | Validation A (GSE104948/54) | 0.601 | 0.455-0.741 | 0.448 | 0.857 |

Diagnostic performance of the 14 candidate genes identified by LASSO regression from urinary single-cell differential expression analysis. The area under the receiver operating characteristic curve (AUC) with 95% confidence interval (CI), sensitivity, and specificity for distinguishing DKD from controls are shown for both the training and independent validation cohorts. Genes are listed in alphabetical order. The three genes selected for the final diagnostic panel (FBP1, PDK4, RHCG) are highlighted in bold.
