# Supplementary material for: A non-invasive urinary diagnostic signature for diabetic kidney disease revealed by machine learning and single-cell analysis
Source: PLoS One. 2026 Jan 2;21(1):e0340096. doi: 10.1371/journal.pone.0340096 (PMC12758759; doi:10.1371/journal.pone.0340096)
Supplement: S2 Table — (DOCX) [file pone.0340096.s008.docx]

**S2 Table. Performance of multivariate diagnostic models built with the three-gene panel (PDK4, RHCG, FBP1).**

| Machine  Learning | Cohort | AUC | 95% CI | Sensitivity | Specificity |
| --- | --- | --- | --- | --- | --- |
|  |  |  |  |  |  |
| Random Forest | Training (GSE96804) | 1.000 | 1.000-1.000 | 1.000 | 1.000 |
|  | Validation B (GSE142025) | 0.977 | 0.933-1.000 | 0.963 | 1.000 |
| Support Vector Machine | Training (GSE96804) | 1.000 | 1.000-1.000 | 1.000 | 1.000 |
|  | Validation B (GSE142025) | 0.996 | 0.984-1.000 | 0.963 | 1.000 |
| XGBoost | Training (GSE96804) | 0.996 | 0.988-1.000 | 1.000 | 0.950 |
|  | Validation B (GSE142025) | 0.994 | 0.979-1.000 | 0.963 | 1.000 |
| Logistic Regression | Training (GSE96804) | 1.000 | 1.000-1.000 | 1.000 | 1.000 |
|  | Validation B (GSE142025) | 0.981 | 0.945-1.000 | 0.963 | 1.000 |

Performance metrics of machine learning models constructed using the three-gene signature (PDK4, RHCG, FBP1). The area under the receiver operating characteristic curve (AUC) with 95% confidence interval (CI), sensitivity, and specificity are reported for the training cohort (GSE96804) and the independent validation cohort B (GSE142025). Sensitivity and specificity were calculated at the optimal probability threshold determined by Youden's index.
